# Comparison Long-term Outcome of Definitive Radiotherapy plus Different Chemotherapy Schedules in Patients with Advanced Nasopharyngeal Carcinoma

**DOI:** 10.1038/s41598-017-18713-z

**Published:** 2018-01-11

**Authors:** Yi-Chun Liu, Wen-Yi Wang, Chih-Wen Twu, Rong-San Jiang, Kai-Li Liang, Po-Ju Lin, Jing-Wei Lin, Jin-Ching Lin

**Affiliations:** 1 Department of Radiation Oncology, Taichung Veterans General Hospital, Taichung, Taiwan; 2 Department of Nursing, Hung Kuang University, Taichung, Taiwan; 3 Department of Nursing, National Taichung University of Science and Technology, Taichung, Taiwan; 4 Department of Otorhinolaryngology, Taichung Veterans General Hospital, Taichung, Taiwan; 5 Faculty of Medicine, School of Medicine, National Yang-Ming University, Taipei, Taiwan; 6 Department of Radiation Oncology, Tungs’ Taichung MetroHarbor Hospital, Taichung, Taiwan; 7 Institute of Clinical Medicine, School of Medicine, National Yang-Ming University, Taipei, Taiwan

## Abstract

Concurrent chemoradiotherapy (CCRT) is the current standard of care for advanced nasopharyngeal carcinoma (NPC). We hypothesize that shifting CCRT to neoadjuvant chemotherapy followed by radiotherapy (NeoCT-RT) is an alternative option. From December 2004 to January 2009, 256 NPC patients with stage II-IVB were treated by either CCRT or NeoCT-RT. All patients received the same dosage and fractionation schedule of RT. After long-term follow-up, 26.8% (34/127) and 23.3% (30/129) of patients who received CCRT and NeoCT-RT respectively, developed a tumor relapse (P = 0.6134). Overall survival (HR = 1.52, 95%CI = 0.91–2.55, P = 0.1532) and progression-free survival (HR = 1.22, 95%CI = 0.75–1.99, P = 0.4215) were similar in both groups. However, acute toxicities during RT period revealed a significant reduction of grade 3/4 vomiting (23% vs. 0%, P < 0.0001), mucositis (55% vs. 16%, P < 0.0001), and neck dermatitis (31% vs. 16%, P = 0.0041) in the NeoCT-RT group, resulting in fewer emergency room visits (10.2% vs. 1.6%, P = 0.0071). Severe treatment-related late toxicity (15% vs. 14%, P = 0.9590) and the occurrence of second malignancy (3.9% vs. 5.4%, P = 0.7887) also showed no differences. We concluded that NeoCT-RT could be an attractive alternative option of CCRT for advanced NPC.

## Introduction

Radiotherapy (RT) is the primary treatment for nasopharyngeal carcinoma (NPC) due to its inherent anatomic constraints and high degree of radiosensitivity. Because NPC is also considered a chemosensitive tumor, a great deal of attention has focused on the combination of RT and chemotherapy in the treatment of locoregionally advanced NPC. In general, there are three different methods in which to incorporate chemotherapy into a curative course of RT: pre (neoadjuvant chemotherapy, NeoCT), during (concurrent chemotherapy, ConCT), and post (adjuvant chemotherapy, AdjCT) irradiation. Most meta-analysis reports have concluded that ConCT is a better opinion than NeoCT or AdjCT^1–4^. The initial concurrent chemoradiotherapy (CCRT) approach was widely adopted as the standard treatment after publication of the Intergroup-0099 study^5^ and recommended in the NCCN guidelines^6^. During recent years, NeoCT has become as an initial treatment choice for advanced NPC, especially in endemic areas with a heavy patient load waiting for RT. In fact, potential benefits of NeoCT approach are reduction of distant metastasis and rapid shrinkage of tumor size that facilitates subsequent RT treatment planning for NPC. The aim of this study is to test the hypothesis whether shifting CCRT to NeoCT-RT could result in similar efficacy in patients with advanced NPC. We analyzed a consecutive 256 NPC patients who finished either CCRT or NeoCT-RT at our hospital with long-term follow-up.

## Results

### Patient characteristics, initial response, and patterns of failure

The consensus guidelines of the initial treatment for advanced NPC in our hospital were either CCRT (according to guidelines from the Western countries) or NeoCT-RT (by our past experiences)^7,8^. This study enrolled 256 patients who received either CCRT (n = 127) or NeoCT-RT (n = 129) as their initial definitive treatment from December 2004 to January 2009. Table 1 outlines the pre-treatment characteristics of the patients. There were no statistically significant differences in terms of age, gender, performance status, pathologic type, T-classification, N-classification, or overall stage. However, patients who received NeoCT-RT had a higher pretreatment plasma EBV DNA levels (median, 962 copies/mL; interquantile range, 163–2381) than those who received CCRT (median, 444 copies/mL; interquantile range, 55–2335). The difference showed borderline significance (P = 0.076).

**Table 1:** Patient characteristics.

Characteristics	CCRT (n = 127)		NeoCT + RT (n = 129)		*P*
	No.	%	No.	%	
Age (years)					0.2822
Range	18–79		15–72		
Median	47.0		46.0		
Gender					0.5323
Male	95	74.8	91	70.5	
Female	32	25.2	38	29.5	
Karnofsky scale					0.7164
>80%	32	25.2	29	22.5	
≤80%	95	74.8	100	77.5	
WHO pathologic type					
Type I	1	0.8	0	0.0	0.2277
Type II	88	69.3	100	77.5	
Type III	38	29.9	29	22.5	
T-classification					0.3216
T1	27	21.3	18	14.0	
T2	31	24.4	35	27.1	
T3	45	35.4	43	33.3	
T4	24	18.9	33	25.6	
N-classification					0.4474
N0	13	10.2	7	5.4	
N1	22	17.3	19	14.7	
N2	65	51.2	74	57.4	
N3	27	21.3	29	22.5	
Overall stage					0.0851
II	20	15.7	9	7.0	
III	60	47.2	66	51.2	
IV	47	37.0	54	41.9	

Abbreviations: CCRT, concurrent chemoradiotherapy; NeoCT, neoadjuvant chemotherapy; RT, radiotherapy; WHO, World Health Organization.

Tumor response, evaluated 2-3 months upon completion of RT revealed that NeoCT-RT showed better complete response rates than CCRT (96.1% vs. 85.0%, P = 0.0019).

Seventy-three of 127 (57.5%) patients in the CCRT group and 82 of 129 (63.6%) in the NeoCT-RT group possessed high-risk factors (P = 0.3853). After finished initial CCRT or NeoCT-RT, patients with high-risk factors agreed to receive post-RT adjuvant tegafur-uracil were no significant differences between the CCRT and NeoCT-RT subgroup (83.6% vs. 91.5%, P = 0.2106).

After a minimal follow-up time of 6 years for surviving patients, 26.8% (34/127) and 23.3% (30/129) of patients who received CCRT and NeoCT-RT respectively, developed a tumor relapse (P = 0.6134). Table 2 demonstrates the detailed patterns of failure. There were no significant differences in local, regional, and distant failures (P = 0.6033, 1.0000, and 0.1809) between the two groups.

**Table 2:** Patterns of failure.

Failure site(s)	CCRT (n = 127)	NeoCT + RT (n = 129)	*P*
T	9	13	
N	2	3	
M	17	8	
T + N	2	1	
T + M	3	3	
N + M	1	1	
T + N + M	0	1	
Sum of any failures	34 (26.8%)	30 (23.3%)	0.6134
Total failures in T	14 (11.0%)	18 (14.0%)	0.6033
Total failures in N	5 (03.9%)	6 (04.7%)	1.0000
Total failures in M	21 (16.5%)	13 (10.1%)	0.1809

Abbreviations: T, nasopharynx; N, neck; M, distant metastasis.

### Survival outcome

**Figure 1:**
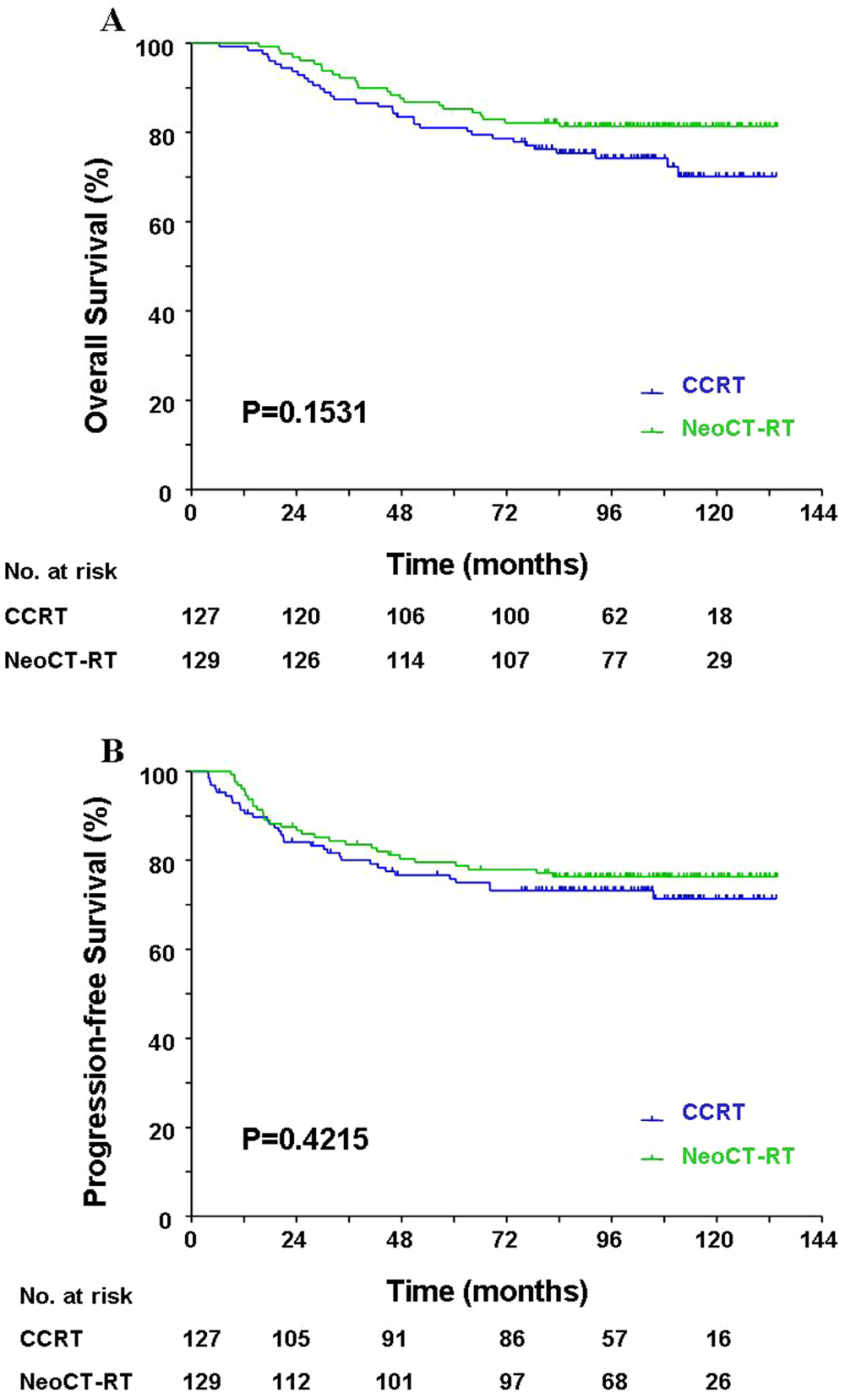
Comparison of the overall survival (**A**) and progression-free survival (**B**) between patients treated with concurrent chemoradiotherapy (CCRT) vs. neoadjuvant chemotherapy + radiotherapy (NeoCT-RT).

**Figure 2:**
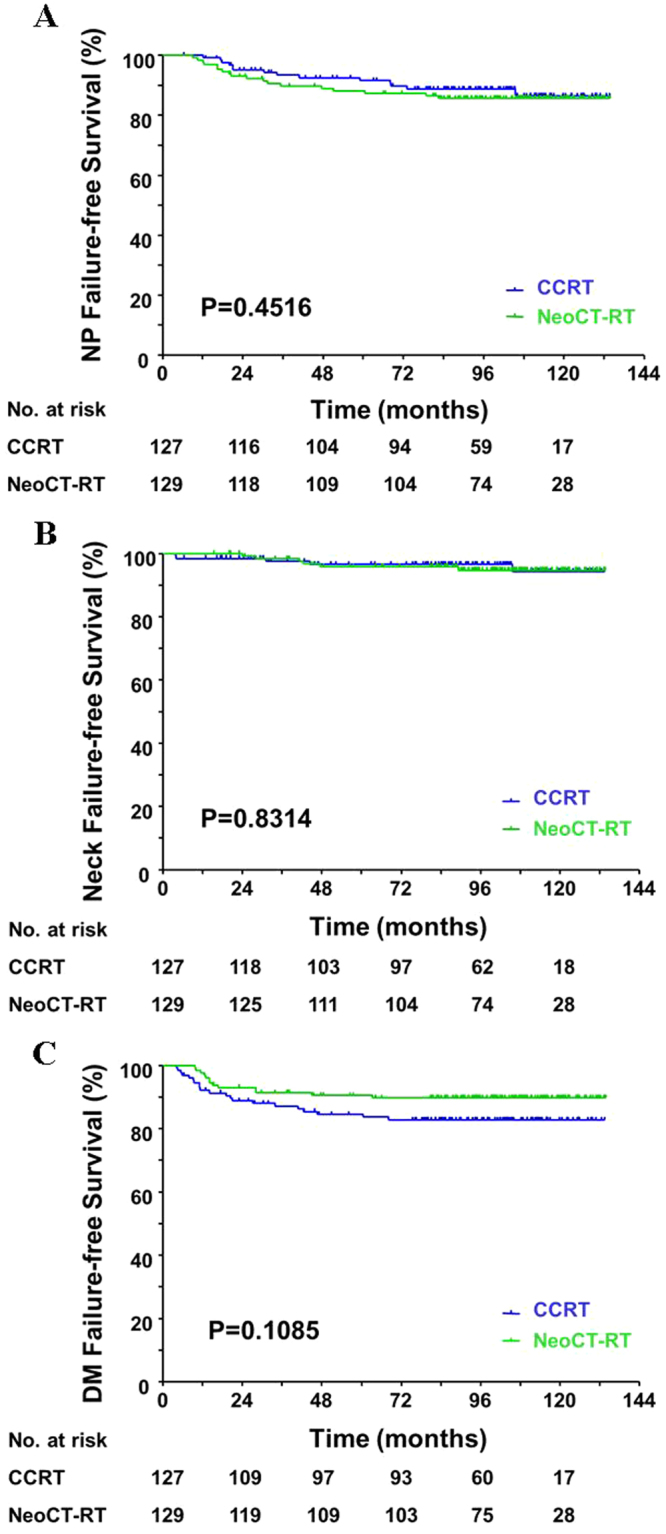
Comparison of the nasopharynx (NP) failure-free survival (**A**), Neck failure-free survival (**B**), and distant metastasis (DM) failure-free survival (**C**) between patients treated with concurrent chemoradiotherapy (CCRT) vs. neoadjuvant chemotherapy + radiotherapy (NeoCT-RT).

The OS (HR = 1.52, 95%CI = 0.91–2.55, P = 0.1531, Fig. 1A) and PFS (HR = 1.22, (95%CI = 0.75–1.99, P = 0.4215, Fig. 1B) were similar in patients who received either CCRT or NeoCT-RT. We also observed no statistically significant differences between the two groups in terms of NPFFS (HR = 0.82, 95%CI = 0.41–1.64, P = 0.4516, Fig. 2A), NFFS (HR = 0.91, 95%CI = 0.28–3.0, P = 0.8814, Fig. 2B), and DMFFS (HR = 1.73, 95%CI = 0.88–3.40, P = 0.1085, Fig. 2C).

### Acute toxicity and tolerance

Acute toxicities during the NeoCT period for the 129 patients who had enrolled in the NeoCT-RT group were well-tolerated and usually mild in their severity (Table 3). Grade 3/4 toxicity included leucopenia (9%), anemia (16%), thrombocytopenia (2%), vomiting (12%), and body weight loss (1%). There was no grade 3/4 mucositis exhibited. Ninety-five percent (122/129) patients were able to complete the scheduled 10 weekly NeoCT. Only 7 patients received less than 10 weekly dose of NeoCT (9 doses in 3 cases, 8 doses in 2 cases, and 7 doses in 2 cases) due to toxicities (n = 2), patient’s refusal (n = 2), acute hepatitis (n = 2), and hyperglycemic hyperosmolar non-ketotic syndrome (n = 1). Ninety-eight percent (126/129) patients were able to receive weekly NeoCT on time, and only 3 patients had one-week delays due to toxicity.

**Table 3:** Acute toxicities during neoadjuvant chemotherapy phase of NeoCT + RT group.

Toxicity	Grade				
	0	1	2	3	4
Leucopenia	35 (27%)	43 (33%)	40 (31%)	11 (09%)	0
Anemia	40 (31%)	36 (28%)	33 (26%)	16 (12%)	4 (3%)
Thrombocytopenia	106 (82%)	13 (10%)	7 (05%)	2 (02%)	1 (1%)
Mucositis	117 (91%)	5 (04%)	7 (05%)	0	0
Vomiting	22 (17%)	62 (48%)	30 (23%)	15 (12%)	0
Weight Loss	51 (40%)	47 (36%)	30 (23%)	1 (01%)	0
Alopecia	95 (74%)	32 (25%)	2 (02%)	0	0

Table 4 compares acute toxicities during the IMRT phase between CCRT and NeoCT-RT. There were no significant differences in hematologic toxicities between the two groups. However, patients who received CCRT had a significantly higher incidence of grade 3/4 vomiting (23% vs. 0%, P < 0.0001), mucositis (55% vs. 16%, P < 0.0001), neck radiation dermatitis (31% vs. 16%, P = 0.0041), and weight loss (6% vs. 0%, P = 0.0068), than those who received NeoCT-RT. One patient in the CCRT refused further RT at 56 Gy. The remaining patients completed the scheduled dose of RT. An RT interruption ≥ 1 week occurred in 16 patients in the CCRT group and in 4 patients in the NeoCT-RT group (12.6% vs. 3.1%, P = 0.0094).

**Table 4:** Acute toxicity during IMRT of both groups.

Grade	0	1-2	3-4	P*
Leucopenia				0.7464
CCRT	29 (23%)	91 (72%)	7 (06%)	
NeoCT-RT	26 (20%)	98 (76%)	5 (04%)	
Anemia				0.0931
CCRT	72 (57%)	52 (41%)	3 (02%)	
NeoCT-RT	44 (34%)	75 (58%)	10 (08%)	
Thrombocytopenia				1.0000
CCRT	121 (95%)	5 (04%)	1 (01%)	
NeoCT-RT	122 (95%)	6 (05%)	1 (01%)	
Mucositis				<0.0001
CCRT	0	57 (45%)	70 (55%)	
NeoCT-RT	6 (05%)	103 (80%)	20 (16%)	
Neck dermatitis				0.0041
CCRT	0	87 (69%)	40 (31%)	
NeoCT-RT	0	109 (84%)	20 (16%)	
Vomiting				<0.0001
CCRT	10 (08%)	88 (69%)	29 (23%)	
NeoCT-RT	117 (91%)	12 (09%)	0	
Weight loss				0.0068
CCRT	2 (02%)	118 (93%)	7 (06%)	
NeoCT-RT	26 (20%)	103 (80%)	0	

Abbreviations: IMRT, intensity-modulated radiotherapy; CCRT, concurrent chemoradiotherapy; NeoCT, neoadjuvant chemotherapy; RT, radiotherapy.

*P-values were calculated by comparing the incidence of the grade 3/4 toxicities between both groups.

The accumulated acute toxicities during the whole treatment course of combined NeoCT and RT arm revealed similar results except for anemia compared with CCRT arm. There were significant less grade 3/4 mucositis (P < 0.0001), radiation dermatitis (P = 0.0025), vomiting (P = 0.0175), and weight loss (P = 0.0294) favoring NeoCT-RT arm. But CCRT arm had less grade 3/4 anemia occurrence (P = 0.0002).

Thirteen of 127 (10.2%) patients in the CCRT group were unable to complete the planned two cycles of concurrent chemotherapy due to toxicity (n = 5) and patient’s refusal (n = 8). The second cycle concurrent chemotherapy needed to be delayed for more than one week in 5 of the patients.

Because the incidence and severity of mucositis and vomiting were higher throughout the CCRT group, these patients had a greater occurrence of elevated serum creatinine levels (23.6% vs. 1.6%, P < 0.0001), hyponatremia (8.7% vs. 0.8%, P = 0.0072), and more additional emergency room visits (10.2% vs. 1.6%, P = 0.0071), than those who were part of the NeoCT-RT group. Other severe events during IMRT included pneumonia in one patient (CCRT group) and hepatitis in 4 patients (1 in CCRT and 3 in the NeoCT-RT group). However, no toxicity resulting in fatalities occurred in either group.

### Late Events

Severe late toxicities occurred in 15% (19/127) patients of the CCRT group and 14% (18/129) patients of the NeoCT-RT group (P = 0.9590). These included hearing impairment (6.3% vs. 4.7%, P = 0.7604), cranial nerve palsy (4.7% vs. 4.7%; P = 1.0000), neck fibrosis (3.1% vs. 3.9%, P = 1.0000), vessels damage (0.8% vs. 1.6%, P = 1.0000), and brachial plexus injury (0.8% vs. 0%, P = 1.0000).

Second malignancies were noted after successful NPC treatment in 5 of 127 patients (2 lungs, 2 breasts, and 1 rectum) who received CCRT and in 7 of 129 patients (3 lungs, 2 tongues, 1 breast, and 1 lymphoma) who received NeoCT-RT. The incidence rates of the second primary were similar in both groups (3.9% vs. 5.4%, P = 0.7887).

## Discussion

RT alone has long been proven to be inferior to combined chemoradiotherapy (CCRT + AdjCT, CCRT, NeoCT-RT) in locally advanced NPC^5,9–22^. The Intergroup-0099 study was the first phase III randomized trial to show both OS and PFS benefits in the CCRT + AdjCT group for advanced NPC^5^. This treatment modality has become the most popularly used standard of care since 1998. Several large phase III randomized trials from endemic areas with a similar design confirmed the findings of the Intergroup study^10–14^. In addition, CCRT alone has also been accepted as another daily practice option with the support of phase III randomized trials^9,15–18^. Three older, randomized trials published more than 10 years ago also demonstrated that NeoCT-RT offered significant improvement in disease-free survival^19^ or relapse-free survival^20–22^, and better, albeit non-significant improvement of OS than the use of RT alone. But no prior randomized studies compared the difference between NeoCT-RT versus CCRT.

Although initial CCRT is the backbone of standard treatment for advanced NPC, there are increase the use of NeoCT before CCRT/RT during the recent ten years. Recently, several new randomized trials were initiated to compare the effect of NeoCT + CCRT versus CCRT alone or NeoCT + CCRT versus CCRT + AdjCT^23–26^. The preliminary results of two large randomized trials (both enrolled about 480 patients) demonstrated that NeoCT + CCRT had survival benefits than CCRT alone. The Taiwan Cooperative Oncology Group by use of a tri-weekly NeoCT (mitomycin-C 8 mg/m^2^ day 1, epirubicin 60 mg/m^2^ day 1, cisplatin 60 mg/m^2^ day 1, 5- fluorouracil 450 mg/m^2^ day 8 and leucovorin 30 mg/m^2^ day 8) and weekly ConCT (cisplatin 30 mg/m^2^) reported a superior disease-free survival but no difference of OS between NeoCT + CCRT and CCRT arms (NCT00201396, unpublished). Using a tri-weekly NeoCT of docetaxel 60 mg/m^2^ day 1, cisplatin 60 mg/m^2^ day 1, 5- fluorouracil 600 mg/m^2^ days 1–5 and a tri-weekly ConCT (cisplatin 100 mg/m^2^), the Sun Yat-sen University Cancer Center, Guangzhou, China obtained better failure-free survival, overall survival, and distant failure-free survival than the CCRT alone^26^. However another two studies containing relatively small sample size (less than 200 patients) revealed that there were no significant differences in terms of PFS^23^ or disease-free survival^24^ between the NeoCT + CCRT and CCRT alone group. The Hong Kong NPC-0501 trial^25^ recruited the largest sample size (n = 801). Unfortunately, no definite conclusions were drawn due to the design of multiple aims being incorporated into one trial exploring 1) chemotherapy sequence: induction vs. adjuvant, 2) chemotherapy regimen: oral capecitabine vs. intravenous 5-FU, and 3) RT schedules: hyperfractionated vs. conventional. The results of this retrospective study revealed that there were no significant differences in terms of various survivals between CCRT and NeoCT-RT.

After carefully re-visiting larger trials which were undertaken during the last ten years, we found that most previous trials had failed to demonstrate a significant improvement of OS in any combined chemoradiotherapy groups. We believe that OS could therefore be similar among the different combinations of initial chemoradiotherapy. Since designing a superior initial chemoradiotherapy regimen (CCRT, NeoCT-RT, or NeoCT-CCRT) to prolong OS would not easily be achieved, selecting a low-toxic regimen may be an option beneficial to the patients.

Although CCRT is the most effective initial treatment for locally advanced NPC, it has been shown to induce a significant increase of acute toxicities in all previous reports. NeoCT-CCRT is also an effective initial treatment modality. However, the acute toxicities displayed during CCRT after NeoCT were usually reported as being similarly severe or even worse than the use of CCRT alone. A recent retrospective study from the Sun Yat-sen University Cancer Center, Guangzhou, China found that the treatment outcomes of the NeoCT + RT and CCRT were similar. However, CCRT led to higher rates of acute and later toxicities for 214 patients with ascending-type NPC^27^. Results of this study confirmed the findings mentioned above with similar sample size (256 patients). In addition, we had a longer follow-up time (median 72.0 vs. 46.8 months) and more uniform dosage of NeoCT or ConCT than the above study. Several unique features of our NeoCT-RT design may have contributed to the significant reduction of acute toxicities when compared with CCRT. First, we used weekly P-FL, as opposed to the conventional tri-weekly PF or TPF during the NeoCT phase. Secondly, we used RT on its own, rather than CCRT after NeoCT, in order to avoid more side effects from CCRT. Thirdly, the dose intensity of our 10 weekly P-FL NeoCT was determined to be adequate for tumor volume reduction. The compliance of our NeoCT is very good- 95% of our patients were able to complete the planned 10-week treatment. The Intergroup study reported only 63% and 55% patients could finish the planned ConCT and AdjCT. All of previous studies using similar design (CCRT + AdjCT) showed similar low compliance rates of ConCT and AdjCT. Although survival analysis showed no statistically significant differences between NeoCT-RT and CCRT in our study, patients who received NeoCT-RT had relatively better DMFFS and OS than those who received CCRT with smaller but not significant P-values (0.1085 and 0.1531).

The major weakness of this study is a retrospective analysis with a relatively small sample size. Of note, the design of combined cisplatin with 5-FU as the concurrent regimen was based on our past experiences^9^. This regimen is different from the standard single agent of cisplatin in most studies. The reported rates on the occurrence of grade 3/4 mucositis during CCRT with tri-weekly high-dose cisplatin were 29% (Intergroup study)^5^, 48.1% (Singapore trial)^10^, 62% (Hong Kong NPC-9901 trial)^9^, and 35.3% (China trial)^26^. Similar rates (31.4% to 48.9%) were observed by changing to a weekly low-dose cisplatin^11,15,28^. We obtained a slightly higher grade 3/4 mucositis (55%) when combined cisplatin and 5-FU was used in this study. The acute toxicity discrepancies probably due to different concurrent chemotherapy regimen will limit our results to apply to the patients who receive concurrent chemotherapy with cisplatin alone. In addition, post-RT AdjCT was recommended to patients with high-risk factors that may contribute to the final treatment results. However, no significant differences between CCRT and NeoCT-RT arms in terms of percentage of the patients possessed high-risk factors (P = 0.3853) and those who actually received post-RT adjuvant tegafur-uracil (P = 0.2106). Furthermore, all meta-analyses revealed no survival differences in NPC patients who received or not received post-RT AdjCT. One of the strengths of this study is adequate follow-up time than most of previous reports. We observed that NeoCT-RT have similar long-term survivals, similar severe late events compared with CCRT, but significant reduction of grade 3/4 acute toxicities and emergency room visits. Results of this study suggest that NeoCT-RT could be an attractive alternative option other than CCRT during daily practice.

## Methods

### Study population

The routine staging workups for newly diagnosed NPC patients in our hospital included a clinical examination of the head and neck region, fiber nasopharyngoscopy with biopsy, magnetic resonance imaging (MRI) with or without computed tomography (CT) scan from the skull base through to the whole neck, chest radiography, whole-body bone scan, abdominal sonography, complete blood count with differentials, platelet count, biochemical profile, EBV antibody titers and plasma EBV DNA concentrations. ^18^F-fluorodeoxyglucose positron emission tomography (PET) scan or PET/CT was usually performed for stage III-IV patients in order to confirm that there was no distant metastasis before entry.

The inclusion criteria for this retrospective analysis were 1) previously untreated patients with stage II-IVB diseases according to the 2002 American Joint Committee on Cancer staging system; 2) no history of prior cancer, except for carcinoma *in situ* of the cervix or non-melanoma cancers of the skin; 3) Karnofsky performance status ≥ 60%; 4) normal liver, renal and bone marrow function; 5) finished curative intensity-modulated radiotherapy (IMRT) with uniform ConCT or NeoCT described below.

### Initial definitive treatment

During the study period, eligible patients were informed the detailed protocols of two different initial definitive chemoradiotherapy (a short 7-week CCRT versus a long course of 10-weekly NeoCT followed by 7-week RT alone). The allocations to different treatment arm were determined by the selection of the patient itself. Physicians in charge started CCRT or NeoCT-RT accordingly within one week after patient decision. This study was approved by the Institutional Review Board of the Taichung Veterans General Hospital, Taiwan, and all procedures were performed according to the Declaration of Helsinki. Written informed consents were obtained from all patients before treatment. Both groups received the same IMRT 70 Gy/35 fractions/7 weeks to the gross tumor volume (primary tumor and positive nodes) plus a 2–4 mm margin. We preferred the use of a hyperfractionated RT with 76.8 Gy (1.2 Gy bid × 64 fractions) for patients suffering from advanced T4 tumors and implemented a 74 Gy by conventional fractionation for those who refused the hyperfractionated RT. The NeoCT consisted of cisplatin 60 mg/m^2^ on day 1 and 5- fluorouracil 2500 mg/m^2^ + leucovorin 250 mg/m^2^ on day 8, repeated every 2 weeks for 5 cycles^7^. The ConCT regimen was cisplatin 20 mg/m^2^/day + 5-fluorouracil 400 mg/m^2^/day, over a 96-hour continuous infusion during the first and 5th week of RT, for two cycles^9^.

### Patient assessments during initial definitive treatment

Complete blood count, platelet count, and body weight were checked and the patients examined every week during the CCRT or NeoCT-RT period. Liver and renal function tests were re-checked once every 3-4 weeks. The acute toxicities were assessed as according to the World Health Organization criteria^29^.

### Post-RT adjuvant chemotherapy

During treatment years, our guideline also recommended post-RT AdjCT with oral tegafur-uracil (each capsule contained 100 mg of tegafur and 224 mg of uracil) via 2 capsules, twice daily for 12 months for patients possessing high-risk factors, such as metastatic nodes > 6 cm, supraclavicular node metastasis, skull base destruction/intracranial invasion plus multiple nodes metastasis, or multiple neck nodes metastasis with one node > 4 cm^30^ and were initiated approximately 3 months after completion of CCRT/NeoCT-RT.

### Response evaluation and follow-up schedule

Re-staging survey, including MRI or CT scan of the head and neck, chest X-ray, abdominal sonography and whole-body bone scan were performed 2-3 months after completion of the initial CCRT or NeoCT-RT to evaluate tumor response, based on the RECIST criteria^31^. These image studies were repeated once every 6–12 months during the first 2 years, and annually thereafter. PET or PET/CT scan was not part of the follow-up routine, but was usually performed if the above-mentioned examinations were deemed equivocal, or if the physicians in charge wanted to obtain a confirmatory diagnosis and evaluation of the extent of recurrence/metastasis before any salvage treatment.

The frequency of post-RT follow-up occurs every 1-2 months in the first year, every 2-3 months in both the second and third years, every 3–6 months in both the fourth and fifth years, and annually thereafter. Flexible nasopharyngoscopy was performed once per 3–6 months during the patient’s follow-up visits.

### Statistical analysis

The primary end-points of this study were progression-free survival (PFS) and overall survival (OS). The secondary end-points were the tumor response rates after the initial definitive treatments, acute and late toxicities, nasopharynx failure-free survival (NPFFS), neck failure-free survival (NFFS), and distant metastasis failure-free survival (DMFFS) between the two groups. PFS was defined as being the time from the first date of definitive treatment, to the time of disease progression or death. OS was defined as the date from which the first day of definitive treatment began, until death of any cause or the date of the last follow-up visit. NPFFS, NFFS, and DMFFS were calculated as being from the first date of definitive treatment until the date of the first occurrence of primary, neck or distant relapse, or the date of the last follow-up visit. An intention-to-treat principle was applied for all patients involved in the analysis.

Patient characteristics and other variables were compared as follows: the Student *t* test was used for continuous variables of the two groups. The Chi-square test was used for category or ordinal variables. The Fisher’s exact test was used when a small sample size existed. Survival curves were estimated through the use of the product-limit method. Survival differences between different subgroups were then analyzed using the log-rank test. All of these statistical tests are two-sided and a *P* value of less than 0.05 is considered statistically significant. Analyses were performed by the use of SAS (Version 8.0; SAS Institute, Inc., Cary, NC).
